# Dose comparison of megavoltage cone‐beam and orthogonal‐pair portal images

**DOI:** 10.1120/jacmp.v8i1.2275

**Published:** 2007-02-28

**Authors:** Lee‐Cheng Peng, Ching‐Chong Jack Yang, Sang Sim, Mitchell Weiss, Alex Bielajew

**Affiliations:** ^1^ Monmouth Medical Center Department of Radiation Oncology New Jersey U.S.A.; ^2^ University of Michigan Department of Nuclear Engineering and Radiological Science Ann Arbor Michigan U.S.A.

**Keywords:** image‐guided radiotherapy (IGRT), megavoltage cone‐beam computed tomography (MV CBCT), electronic portal image device (EPID), dose comparison

## Abstract

The technique of megavoltage cone‐beam computed tomography (MV CBCT) is available for image‐guided radiation therapy to improve the accuracy of patient setup and tumor localization. However, development of strategies to efficiently and effectively implement this technique or to replace the current orthogonal portal images technique remains challenging in the clinical environment. It is useful to compare the difference in absorbed dose between the MV CBCT technique and the orthogonal portal images technique, the current standard practice for treatment verification. Our study analyzed the doses generated from these two imaging techniques for six treatment sites (pelvis, abdomen, lung, head and neck, breast, prostate). The analysis was made by simulating the MV CBCT technique with an arc beam and a beam‐on time of 9 monitor units (MUs), and the orthogonal pair technique with a double‐exposure anterior–posterior and lateral pair and a beam‐on time of 4 MUs. The results are presented as dose per MU (cGy/MU) and absolute dose (cGy). The isocenter doses, integral doses, maximum doses, and mean doses to tumor and critical organs, and the two‐dimensional isodose distributions and dose–volume histograms of each critical organ were investigated. The absolute dose difference between MV CBCT and orthogonal pair at the isocenter was 4.02±0.59 cGy. Major differences were seen between the two techniques in critical organs whose locations are away from the tumor. These organs, such as the contralateral breast (difference: 0.17±0.10 cGy/MU) and lung (difference: 0.15±0.20 cGy/MU), receive a higher dose from MV CBCT images than from orthogonal portal images. Additionally, higher doses and larger dose areas involving more normal tissues were observed for MV CBCT images than for orthogonal portal images in our analysis methodology, which used 200 beam projections delivered from various angles for the MV CBCT simulation and from just two perpendicular angles for the orthogonal pair simulation. In our selected clinical cases, the high‐dose area from the orthogonal pair technique was always located inside the tumor; with MV CBCT, the high‐dose area will most likely be outside the tumor. Therefore, the potentially higher doses to critical organs from MV CBCT images should be properly analyzed to ensure that they do not exceed the tolerance dose when therapy is delivered using that technique. On the other hand, to obtain good image quality, the higher MUs with MV CBCT images may be necessary. The absorbed dose for the tumor and for other critical organs should be calculated accordingly in the treatment plans. Images by MV CBCT are a great tool for three‐dimensional verification of patient treatment position. The trade‐off is that the MV CBCT technique for patient treatment verification might have a higher chance of increasing the dose to normal tissue during image acquisition.

PACS number: 87.53.Oq

## I. INTRODUCTION

The importance of accurate radiation treatment delivery has been discussed theoretically^(^ ^1^ ^,^ ^2^ ^)^ and demonstrated clinically.^(^ ^3^ ^,^ ^4^ ^)^ The consequence of missing the tumor in radiation therapy is a lowered tumor control probability, which might translate into a lower survival rate. The probability of normal‐tissue complications are also increased. Thus, verification of patient treatment position is a crucial aspect of radiation therapy.

In current clinical practice, variations in patient setup and organ motion are the two most important limiting factors to radiation treatment precision. Traditionally, orthogonal portal film images have been compared with the corresponding simulator image or a digitally constructed radiograph to verify the beam isocenter position and beam angle and shape. This comparison usually occurs at the beginning of the treatment, and it is repeated at regular intervals throughout the treatment course. However, the filming procedure is time consuming and labor intensive.

Recently, electronic portal imaging devices (EPIDs) have gained in popularity as the device of choice for patient setup verification.^(^ ^5^ ^,^ ^6^ ^)^ This popularity is attributable mainly to the high‐sensitivity detectors used in EPID, which, in a busy clinic, provide an efficient and effective method for accurately determining radiation field placement.

However, regardless of whether portal imaging is film‐based or EPID‐based, one of the shortcomings of the technique is that it examines only the location of the bony anatomy. It does not provide information on the tumor or other soft tissues. Current advanced treatment techniques such as serial tomotherapy and intensity‐modulated radiotherapy generate a spatially and temporally complex variation of intensities with a large intensity gradient. The accuracy of treatment positioning therefore becomes an even more critical issue. To localize the tumor and critical organs more accurately, image‐guided radiation therapy has been introduced.

One form of image‐guided radiation therapy uses cone‐beam computed tomography (CBCT), a technique by which the patient three‐dimensional (3D) image set is reconstructed from a series of two‐dimensional (2D) projection images acquired immediately before the treatment. Two CBCT techniques have been developed: kilovoltage (kV) CBCT and megavoltage (MV) CBCT.^(^ ^7^ ^,^ ^8^ ^)^

The kV CBCT system consists of a kV X‐ray tube and a radiographic detector mounted on the gantry of a medical linear accelerator. The MV CBCT system uses the existing EPID, making it more cost effective. Moreover, because the same radiation source is used for both imaging and therapy, no cross‐calibration procedure is needed because the imaging geometry is the same as the treatment geometry. However, clinical implementation of MV CBCT has been challenging because of the relatively large dose required to achieve acceptable image quality.^(^ ^7^ ^–^ ^9^ ^)^

Using a large‐area cesium iodide detector viewed by television camera, Moslesh–Shirazi et al. required 40 cGy to produce images with reasonable contrast and spatial resolution.^(^ ^10^ ^,^ ^11^ ^)^ The first generation of MV CBCT with amorphous silicon detectors needed high (50–200 cGy) doses^(^ ^7^ ^,^ ^12^ ^)^ to achieve good image quality. Advances in amorphous silicon technologies in the past few years have made low‐dose MV CBCT feasible through the use of fast, sensitive, and high‐spatial‐resolution detectors.^(^ ^13^ ^,^ ^14^ ^)^ Simpson et al.^(15)^ developed a prototype megavoltage CT scanner with 110 projections and delivered a dose of approximately 12 cGy. Recently, Seppi et al.^(16)^ obtained high‐quality megavoltage CT images using 16 cGy (360 projections×0.046 cGy/projection), and Pouliot et al.^(13)^ also reported reasonable image quality acquired with 5–15 cGy (180 projections×0.027−0.083 cGy/projection).

In accordance with the ALARA (“as low as reasonably achievable”) principle in radiation protection, doses from MV CBCT images should be reduced as far as reasonably possible. As a reference for comparison, the dose delivered by the traditional orthogonal portal images technique,^(^ ^17^ ^–^ ^19^ ^)^ employing either film or EPID, is deemed clinically acceptable. The purpose of the present study was to (1) obtain dose information for six different treatment sites by simulating and calculating the delivered doses from the MV CBCT and orthogonal pair techniques, and (2) report for the two imaging techniques the dose per monitor unit (cGy/MU) and the absolute dose (cGy) at the isocenter, the integral dose, the maximum dose in the patient, and the average doses to the tumor and critical organs.

## II. MATERIALS AND METHODS

We calculated the doses to the patient resulting from the orthogonal pair and the MV CBCT imaging techniques, both based on a 6‐MV Oncor linear accelerator (Siemens Medical Solutions, Malvern, PA) equipped with an amorphous silicon flat panel (AG‐9). All calculations were done on a Pinnacle 3D treatment planning system (Version 7.4f, Philips Medical Systems, Andover, MA).

For the orthogonal pair technique, an anterior–posterior (AP) and a lateral (LAT) field were created, each with a field size of 20×20 cm and a beam‐on time of 3 MUs (the standard practice in our clinic). Thus, a total of4 MUs were used for the AP/LAT orthogonal pair.

**Figure 1:**
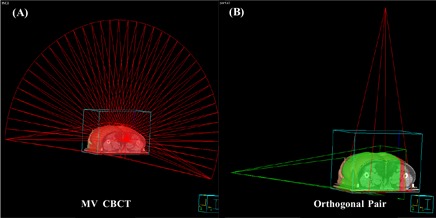
(A) The room's eye view of a megavoltage cone‐beam computed tomography image, created by delivering an arc beam from 270 degrees to 110 degrees, clockwise direction. (B) The room's eye view of orthogonal portal images with 0‐degree (anterior–posterior) and 90‐degree or 270‐degree (lateral) acquisition as an orthogonal pair.

For the MV CBCT technique, a 200‐degree arc beam was created from 270 degrees to 110 degrees (International Electrotechnical Commission scale) in a clockwise direction as recommended by the manufacturer. For the purpose of the dose calculation, the arc beam was divided into 200 fixed subfields at 1‐degree intervals, and doses were calculated for each sub‐field. The total beam‐on time for the arc was set to 9 MUs, as suggested by Pouliot et al.^(^ ^13^ ^,^ ^20^ ^)^ to achieve good image quality for head‐and‐neck treatment. To achieve a fair dose comparison with the orthogonal pair technique, the field size was also set to 20×20 cm. The room views of the MV CBCT and orthogonal pair techniques are shown in Fig. 1 (panels A and B respectively).

To quantify and compare the doses to the tumor and the surrounding critical organs resulting from the MV CBCT and orthogonal pair techniques, 30 patients representing six different treatment sites (pelvis, abdomen, lung, head and neck, left breast, and prostate) were analyzed in our study. Data from 5 patients for each treatment site were used to calculate the means and standard deviations. The calculated doses were the integral dose, maximum dose to the patient, dose at the isocenter, and mean dose to the tumor and to each critical organ. Table 1 lists the critical organs for each treatment site used in our study. For all patients, the contours of the tumor and the critical organs were drawn by the same physician.

**Table 1:** Critical organs for each treatment site, where GTV is the gross tumor volume and CTV is the clinical tumor volume

Pelvis	Abdomen	Lung	Head and neck	Left breast	Prostate
Max dose	Max dose	Max dose	Max dose	Max dose	Max dose
Isocenter Integral dose	Isocenter Integral dose	Isocenter Integral dose	Isocenter Integral dose	Isocenter Integral dose	Isocenter Integral dose
GTV/CTV Bladder Rectum Kidney	GTV/CTV Spinal cord Lung Kidney Liver Spleen	GTV/CTV Spinal cord Lung Heart Esophagus	GTV/CTV Spinal cord Brainstem Parotid Mandible	GTV/CTV Spinal cord Lung Heart Contralateral breast	GTV/CTV Bladder Rectum Bowel

The calculated doses from the MV CBCT and orthogonal pair techniques can be calculated in two ways—namely, dose per MU (cGy/MU) and absolute dose with specific MU values. For estimating the dose to the patient, the absolute dose is of interest. However, because the actual MUs used in MV CBCT may vary with the treatment site and the imaging protocols adopted in each institution, reporting the dose per MU is also useful. Dose per MU provides a means for easily scaling the calculated dose results to other MU settings.

## III. RESULTS

For each of the 30 patients included in this study, the doses delivered by the MV CBCT and orthogonal pair techniques were calculated and analyzed. Table 2 shows the dose per MU for the two techniques for all six treatment sites. For each treatment site, 5 patients were included in the calculation of the means and standard deviations. The dose per MU was calculated by dividing the calculated absolute dose for each imaging technique by its corresponding MUs: MV CBCT by 9 MUs, and orthogonal pair by 4 MUs.

**Table 2:** Value and standard deviation of the relative dose differences (cGy/MU) between megavoltage cone‐beam computed tomography (MV CBCT) images and orthogonal portal images in six treatment sites for tumor and 14 critical organs, where GTV is the gross tumor volume and CTV is the clinical tumor volume

	Pelvis		Abdomen		Lung	
	MV CBCT	Portal	MV CBCT	Portal	MV CBCT	Portal
Max dose	1.15±0.02	1.43±0.01	1.14±0.06	1.36±0.06	1.16±0.01	1.25±0.1
Isocenter	0.74±0.04	0.78±0.03	0.78±0.06	0.84±0.05	0.84±0.02	0.83±0.08
Integral dose	0.58±0.12	0.73±0.07	0.54±0.12	0.67±0.19	0.62±0.04	0.60±0.04
GTV/CTV	0.72±0.07	0.76±0.08	0.80±0.07	0.84±0.06	0.88±0.06	0.89±0.05
Rectum	0.96±0.02	0.98±0.03				
Bladder	0.62±0.15	0.69±0.13				
Kidney	0.87±0.06	0.9±0.16	0.43±0.28	0.42±0.29		
Liver			0.65±0.07	0.90±0.14		
Spleen			0.40±0.21	0.29±0.25		
Lung			0.59±0.16	0.88±0.09	0.69±0.22	0.54±0.20
Esophagus					0.84±0.04	0.79±0.04
Heart					0.72±0.21	0.69±0.21
Spinal cord			0.43±0.19	0.55±0.23	0.61±0.11	0.58±0.10
	Head and neck		Left breast		Prostate	
	MV CBCT	Portal	MV CBCT	Portal	MV CBCT	Portal
Max dose	1.16±0.09	1.18±0.09	1.16±0.03	1.23±0.03	1.12±0.03	1.22±0.05
Isocenter	1.02±0.01	1.03±0.01	0.81±0.13	0.84±0.11	0.71±0.03	0.72±0.03
Integral dose	0.67±0.07	0.66±0.08	0.57±0.04	0.57±0.06	0.54±0.04	0.54±0.04
GTV/CTV	0.85±0.18	0.87±0.17	1.05±0.05	1.08±0.02	0.71±0.04	0.70±0.06
Rectum					0.70±0.27	0.69±0.26
Bladder					0.64±0.16	0.65±0.13
Bowel					0.56±0.07	0.57±0.08
Lung			0.41±0.04	0.37±0.10		
Heart			0.84±0.07	0.81±0.06		
Contralateral breast			0.40±0.06	0.23±0.05		
Spinal cord	0.80±0.15	0.79±0.14	0.40±0.01	0.60±0.05		
Brainstem	0.31±0.28	0.31±0.28				
Parotid gland	0.58±0.40	0.65±0.49				
Mandible	1.05±0.01	1.05±0.00				

Comparing the two techniques, the integral dose and the dose to the isocenter were similar for all treatment sites. However, the maximum dose to the patient showed greater variation for all sites except the head‐and‐neck site. The thicker treatment sites, such as the pelvis and the abdomen, showed larger differences (1.15 cGy/MU vs. 1.43 cGy/MU and 1.14 cGy/MU vs. 1.36 cGy/MU respectively). Furthermore, the differences in the mean dose to critical organs were larger for organs whose locations were farther from the tumor or the isocenter—for example, normal lung (0.59 cGy/MU vs. 0.88 cGy/MU) and contralateral breast (0.40 cGy/MU vs. 0.23 cGy/MU). In contrast, the differences in the dose per MU to the tumor [gross tumor volume (GTV) / clinical tumor volume (CTV)] or to the critical organs located closer to the tumor were very small (overall average: 0.04±0.016 cGy/MU) between the MV CBCT and orthogonal pair techniques.

Table 3 shows the absolute dose difference for the six treatment sites. Because of the greater MUs employed in the MV CBCT technique, the absolute dose from the MV CBCT technique was higher than that from the orthogonal pair technique. The dose difference ranged from 1.46±0.45 cGy to 5.73±0.43 cGy for various organs.

**Table 3:** Value and standard deviation of the absolute dose difference between megavoltage cone‐beam computed tomography (MV CBCT) images with a total of 9 monitor units (MUs) and portal images with a total of 4 MUs in six treatment sites for tumor and 14 critical organs, where GTV is the gross tumor volume and CTV is the clinical tumor volume

	Pelvis	Abdomen	Lung	Head and neck	Left breast	Prostate
Max dose	4.67±0.17	4.81±0.44	5.46±0.35	5.73±0.43	5.56±0.39	5.17±0.14
Isocenter	3.65±0.22	3.70±0.57	4.23±0.24	5.11±0.08	3.94±0.81	3.51±0.22
Integral dose	2.27±0.93	2.22±0.41	3.22±0.19	3.41±0.35	2.82±0.39	3.63±0.12
GTV/CTV	3.20±0.45	3.83±0.67	4.38±0.50	4.98±0.95	5.21±0.49	2.71±0.22
Rectum	4.67±0.15					3.54±1.39
Bladder	2.87±0.60					3.17±0.91
Bowel						3.62±0.34
Kidney	4.20±2.58	2.51±1.60				
Liver		2.22±0.51				
Spleen		2.39±0.86				
Lung		1.77±1.79	4.02±124		3.89±0.36	
Esophagus			4.40±0.37			
Heart			3.69±1.09		4.33±0.650	
Contralateral breast					2.65±0.49	
Spinal cord		1.65±0.96	3.14±0.61	4.01±0.77	1.46±0.45	
Brain Stem				1.57±1.39		
Parotid				2.60±1.68		
Mandible				5.52±0.07		

**Figure 2:**
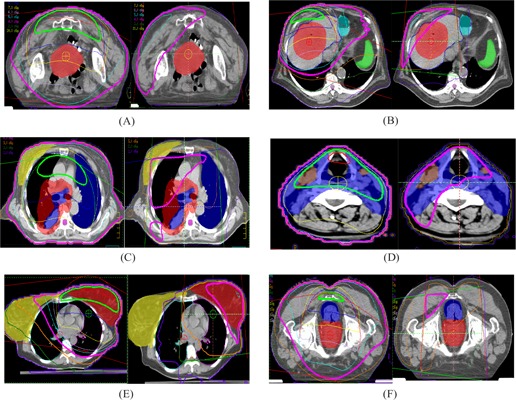
The two‐dimensional absolute dose distribution of transverse central slice is evaluated in various treatment sites—(A) pelvis, (B) abdomen, (C) lung, (D) head and neck, (E) breast, and (F) prostate—using megavoltage cone‐beam computed tomography images at 9 MUs (left panels) and orthogonal portal images at 4 MUs (right panels).

**Figure 3:**
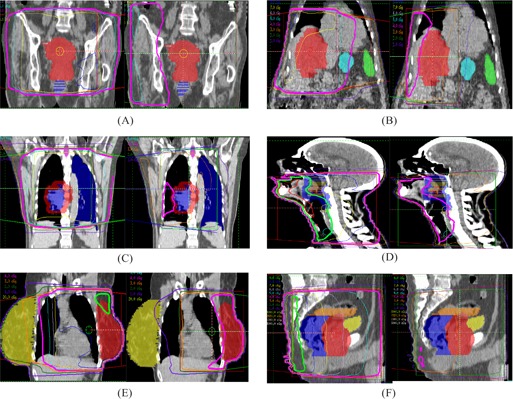
The two‐dimensional absolute dose distribution of sagital/coronal central slice is evaluated in various treatment sites—(A) pelvis, (B) abdomen, (C) lung, (D) head and neck, (E) breast, and (F) prostate—using megavoltage cone‐beam computed tomography images at 9 MUs (left panels) and orthogonal portal images at 4 MUs (right panels).

Fig. 2 shows the 2D absolute dose distributions on the transverse slice that contains the isocenter for the six treatment sites: (A) pelvis, (B) abdomen, (C) lung, (D) head and neck, (E) left breast, and (F) prostate. The left and right panels show the dose distributions from, respectively, the MV CBCT technique and the orthogonal pair technique. The tumor and the critical organs are shown in different colors, and the isocenter is located at the center of the tumor. Fig. 3(A–F) shows the dose distributions on the sagital/coronal slice.

In Figs. 2 and 3, the isodose lines corresponding to the given MUs (9 cGy for MV CBCT and 4 cGy for orthogonal pair) are displayed with medium thickness. Compared with the orthogonal pair technique, the area covered by the 4‐cGy isodose line of the MV CBCT technique is larger, including more volume of the critical organs. With the orthogonal pair technique, the isocenter is located at the center of the tumor, thereby contributing higher dose to the tumor, but lesser doses to the normal tissue away from the tumor. Moreover, the high‐dose area is located at the proximal corner of the rectangular area intersected by the two orthogonal beams. In contrast, because of the anterior arc, the high‐dose area in the MV CBCT technique is located anterior to the anatomy, where it will contribute more dose to the more anterior critical organs such as the normal lung and the contralateral breast.

At the max dose point, the dose per MU was similar between the two techniques, with an average difference of −0.13±0.096 cGy/MU. However, the difference in absolute dose was greater, averaging 5.23±0.43 cGy [10.35±0.43 cGy for MV CBCT (9 MUs) and 5.12±0.11 cGy for orthogonal pair (4 MUs)] over all of the patients.

**Figure 4:**
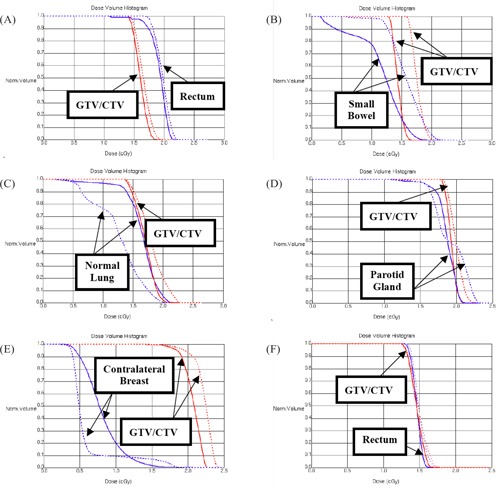
The dose–volume histogram (DVH) of tumor [gross tumor volume (GTV) / clinical tumor volume (CTV)] and all critical organs is evaluated in various treatment sites—(A) pelvis, (B) abdomen, (C) lung, (D) head and neck, (E) breast, and (F) prostate—using megavoltage cone‐beam computed tomography images (solid line) and orthogonal portal images (dashed line) at the same monitor unit value (2 MUs total).

In Fig. 4, the dose–volume histograms (DVHs) are shown for the six treatment sites: (A) pelvis, (B) abdomen, (C) lung, (D) head and neck, (E) breast, and (F) prostate. The doses were calculated with 2 MUs for both techniques. The results of the MV CBCT and orthogonal pair techniques are displayed as solid lines and dashed lines respectively. Red and blue lines show tumor (GTV/CTV) and critical organs separately. For the pelvis, prostate, and head‐and‐neck sites, the DVH difference between the two techniques is small. For the breast, lung, and abdomen treatment sites, the DVH difference is greater, especially for large‐volume critical organs located away from the tumor. Therefore, the dose distribution and absolute dose received by the critical organs in specific treatment sites should draw more attention when MV CBCT images are used in clinical treatment.

## IV. DISCUSSION

Patient motion or setup error during the treatment course can sometimes lead to a geometric miss. Verifying the treatment field at the beginning of treatment and at regular intervals during the course is therefore important. Weekly port filming is a standard procedure used to verify treatment delivery and thus to ensure the correct delivered dose.

For the past 20 to 30 years, regular filming technique with a wet processor has been used for portal images. Digital imaging with phosphor plates or EPIDs has been gaining popularity and has the potential to become the “gold standard” for patient setup verification. As the treatment technique becomes more complex, more precise patient positioning is needed. As a result, 3D imaging techniques such as CBCT, which reconstructs 3D volumes from a series of 2D projection images, have recently been developed. To achieve good image quality, the CBCT technique requires at least 180 projections.^(14)^ The value of the delivered MUs and the number of projections for MV CBCT imaging are therefore the most important factors that should be considered in the clinical application of that technique as compared with the current orthogonal portal images technique.

Many studies have investigated the possibility of using standard CT reconstruction techniques and specialized megavoltage photon detectors with a modest radiation dose (15–20 cGy) to obtain a tomographic image of the patient during treatment.^(^ ^13^ ^,^ ^14^ ^,^ ^16^ ^)^ Given the calculations in the present study, the dose to the isocenter using MV CBCT with a 200‐degree arc and 9 MUs was 6.69±0.05 cGy for pelvis and prostate, 7.42±0.13 cGy for breast and lung, and 9.18±0.01 cGy for head and neck. The absorbed dose varied with the treatment site because of variations in site thickness. However, compared with the portal images technique, the dose to the isocenter from one orthogonal pair using 4 MUs was 3.00±0.03 cGy for pelvis and prostate, 4.67±0.033 cGy for breast and lung, and 4.12±0.0054 cGy for head and neck. To reduce the total dose or the dose outside the treatment volume, the clinical application of MV CBCT should be investigated by improving the sensitivity and efficiency of detectors in the EPID, adjusting the size of the field of view, and performing a limited number of projections. In addition, the frequency of use of MV CBCT should follow the ALARA principle in radiation protection as dictated by professional judgment and institutional practice.

**Figure 5:**
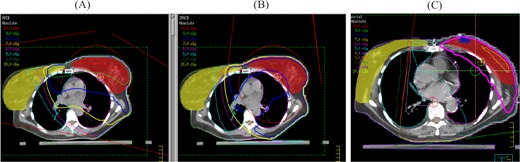
The two‐dimensional absolute dose distribution of a central slice is evaluated in three different beam‐delivery methods with 6 MUs. (A) Megavoltage cone‐beam computed tomography images with 200 projections; (B) megavoltage cone‐beam computed tomography images with 360 projections (from 0 degrees to 359.9 degrees in a clockwise direction); (C) orthogonal pair images.

The significance of the additional MV CBCT dose and the location of the high‐dose area could be questioned. Because the MV CBCT image is reconstructed from many projection images, an arc beam of at least 180 degrees is required. The 2D dose distribution on each slice therefore varies depending on the beam angles spanned by the arc, as shown in Fig. 5. In Fig. 5, a wider arc angle in MV CBCT produces a more uniform dose distribution, but delivers more dose to normal organs such as the contralateral breast or normal lung than does the orthogonal portal images technique (although the difference in the integral dose is small).

The high‐dose area in orthogonal portal imaging is always located inside the tumor or close to it, so that the extra dose will not be a significant issue in clinical treatment. However, with MV CBCT imaging, the high‐dose area may be inside normal critical organs located away from the tumor. The effect could be significant and could possibly lead to secondary malignancies, depending on the threshold dose of the irradiated organs. If high doses are necessary for verification of patient treatment location, then the extra dose should be calculated and evaluated in treatment planning to ensure that it does not exceed the tolerance dose of sensitive organs. The comparisons of the MV CBCT and orthogonal pair techniques in the present study show significant differences in the DVHs seen in abdomen, lung, and breast sites, but almost the same values for DVHs in the pelvis and prostate. The difference is greater for critical organs whose locations are far away from the tumor than for those closer to the tumor. The dose from MV CBCT should be included in the dose–volume histogram for the plan evaluation.

## V. CONCLUSION

Our study calculated doses, dose distributions, and DVHs resulting from both the MV CBCT and orthogonal pair techniques were for six treatment sites. The calculation for the orthogonal pair technique was based on 4 MUs and that for MV CBCT was based on 9 MUs. The latter was considered to be feasible for routine clinical application—providing good image quality while keeping the dose to the patient relatively low.

From our analysis, the relatively high‐dose regions generated by MV CBCT occur inside critical organs and tend to be larger than those generated by the orthogonal pair technique. Radiation‐induced secondary neoplasm is always a concern in radiation therapy. Because of the potential biologic effects caused by the small dose from the imaging process, the extra dose burden to the critical structures should be monitored carefully.

Our study provides a quantitative analysis on the extra radiation burden caused by current verification procedures. It suggests that the number of projections and the total MU value are the most important factors when the MV CBCT technique is used in clinical application. The pros and cons of using MV CBCT for patient treatment verification should be examined to balance the risks and benefits.

## ACKNOWLEDGMENTS

The authors gratefully thank Dr. Chen‐Shou Chui for his comments and revision of the manuscript and Mr. Hensen Chen for his initial idea.
